# Information available on the internet about pain after orthognathic surgery: A careful review

**DOI:** 10.1590/2176-9451.19.6.086-092.oar

**Published:** 2014

**Authors:** Matheus Melo Pithon, Elinailton Silva dos Santos

**Affiliations:** 1 State University of Southwestern Bahia, Department of Orthodontics, Adjunct professor, Department of Orthodontics, State University of Southwestern Bahia (UESB); 2 UESB, Undergraduate student in Dentistry, UESB

**Keywords:** Orthognathic surgery, Pain, Internet, Information

## Abstract

**OBJECTIVE:**

Investigate the quality of data available on the internet with respect to pain after orthognathic surgery.

**METHODS:**

A careful search was conducted on the Internet in December, 2012. The most accessed websites browsers were employed for research using the terms: "pain" and "orthognathic surgery" together. The first 30 results of each portal were examined, and after applying the exclusion criteria, 29 sites remained. All remaining websites went through an evaluation process with online tools that investigated the quality, level of reading, accessibility, usability and reliability.

**RESULTS:**

Assessment criteria outcomes were considered unfavorable. Texts were considered difficult to read with inappropriate language for the general public. The mean global validation for the 29 websites of the LIDA instrument was 65.10, thereby indicating a structure of medium quality.

**CONCLUSION:**

Information about post-orthognathic surgery pain available on the internet is poorly written and unreliable. Therefore, candidates for orthognathic surgery must seek information from specialists who, in turn, should indicate reliable sources.

## Abstract (pt)

**OBJETIVOS:**

verificar a qualidade dos dados disponíveis na internet a respeito da dor após cirurgia ortognática.

**MÉTODOS:**

para a realização desse estudo, foi feita busca criteriosa na internet, no mês de dezembro de 2012. As páginas de busca mais populares foram utilizadas para pesquisa, utilizando os termos "dor" e "cirurgia ortognática" inseridos juntos. Foram averiguados os 30 primeiros resultados de cada portal e, após aplicar os critérios de exclusão, restaram 29 sites, que passaram pelo processo de avaliação em ferramentas *online,* para investigar sua qualidade, nível de leitura, acessibilidade, usabilidade e confiabilidade.

**RESULTADOS:**

os resultados foram desfavoráveis para os critérios avaliados. O nível de leitura dos textos foi considerado difícil de ser lido, apesar de as informações terem como alvo o público geral. Para as 29 páginas da *web*, a média global da validação pelo instrumento LIDA foi de 65,10, indicando uma estrutura avaliada como tendo qualidade média.

**CONCLUSÃO:**

as informações na internet sobre dor pós-cirurgia ortognática são mal escritas e não confiáveis. Por isso, candidatos à cirurgia ortognática devem buscar informações de especialistas, que, por sua vez, devem indicar fontes confiáveis.

## INTRODUCTION

Patients have increasingly sought health information on the internet.^1^^,^ 2 Researches show that over 70% of American adults seek health advice in the world computer network.^3^^-^ 7 The growth in the use of the World Wide Web for this matter functions as a mean of education and prevention^8^ in the health care of children,^9^ adolescents and adults.^10^ In this context, internet users have also sought relevant information about dental procedures^6^ with the intention to find out about patients' experiences and professionals' opinions with regard to a certain procedure.

When orthodontic treatment involves interaction with orthognathic surgery, the most frequent questions are about post-operative pain and discomfort.^11^^,^ 12 Some authors assert that esthetics, psychosocial factors^13^ and relief of functional problems are the main reasons leading patients to seek orthognathic surgery.^14^ On the other hand, they commonly give up due to fear of post-surgical pain.^15^

Based on the foregoing statements and due to the fact that an increasing number of patients use the internet to research health issues including orthognathic surgery, it is necessary to verify the nature quality of information available. Therefore, the present study aims at carefully assessing the information available on the internet about pain after orthognathic surgery.

## MATERIAL AND METHODS

## Research methods

**Figure 1.**
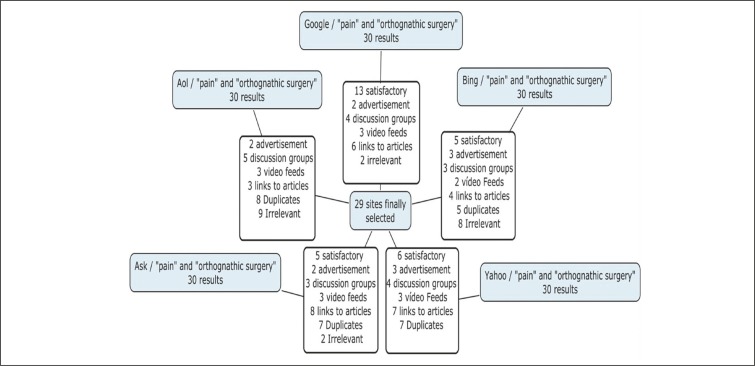
Flow diagram of the selection process.

A research was conducted on the internet in December, 2012. The five major browsers providing access to popular searching tools were used: Google (www.google.com), Bing (www.bing.com), Yahoo! (www.yahoo.com), Ask (www.ask.com) and AOL (www.aol.com). The following terms were used: "pain" and "orthognathic surgery". These terms were used together to simulate the typical on-line research of a lay patient. The exclusion criteria were as follows: Websites for promotional products, discussion groups, duplicates, video feeds and links to scientific articles (Fig 1). Author's name, profession and type of information of all eligible internet portals were also retrieved.

## Quality assessment

Precision may be defined as the level of agreement between the information disclosed and the best evidence generally accepted in clinical practice. In this item, guidelines based on scientific evidence, textbooks and primary literature were used as source of reference to assess the precision of information provided by the websites.^16^ Three criteria were used: duration, etiology and pain management. Therefore, the following classification was used to evaluate precision: **Score 0** = the subject of pain was not discussed; **Score 1** = only one criterion was discussed; **Score 2** = two criteria were discussed; **Score 3** = all three criteria were discussed with important omissions; **Score 4** = all three criteria were discussed with minor omissions; **Score 5** = complete discussion. The end results were converted into percentages so as to render presentation of combined results easier.

## Readability

It is measured by the reading skill an individual needs in order to understand what is written. Flesch Reading Ease Scale (FRES) was used to assess readability. FRES, average sentence length (ASL) and average number of syllables per word (ASW) are interconnected by means of the following equation: FRES = 206.835 - (1.015 x ASL) - 84.6 x ASW. The result may vary from 0 to 100. The higher the result is, the easier the text is to read. Texts scoring between 90 and 100 are considered easily understandable by a pupil in the fifth grade of primary school. Scores between 60 and 70 may be easily understood by pupils in the eighth and ninth grades of primary school. Lastly, scores not greater than 30 account for a reading level of university graduates or undergraduates. To increase the veracity of this study, a passage between 200-500 words was extracted from each website, and then copied and pasted in an online FRES calculator program (www.readabilityformulas.com/free-readability-formula-tests.php).

Complementary quality analysis was obtained by means of LIDA instrument (LIDA instrument, Version 1.2, Minervation Ltd, Oxford, United Kingdom). This validation tool was created to assess the architecture and content of health care websites, in which three distinct areas are assessed: accessibility, usability and reliability. Accessibility score is calculated when one fills out the address of the website in a personalized web platform (www.minervation.com/mod/LIDA). Subsequently, a questionnaire comprising nine questions appear to assess usability and reliability. Responses are graded from 0 to 3 (0: never; 1: sometimes; 2: most of the times; 3: always). This software produces final percentages that account for high, medium or low quality. LIDA score, which is the mean value of the three sub-scores, indicates the general classification of the website.

## RESULTS

## Research results

Research methods retrieved 150 websites for analysis of relevance. Meticulous initial selection led to a total of 29 websites that met the demands of the study (Table 2). Apparently, 12 advertisements, 19 discussions, 14 video feeds, 28 links to scientific articles, 27 duplicates, and 21 web pages with irrelevant content were excluded from the last phase of classification (Fig 1).

**Table 1.** Questions of LIDA instrument used to assess usability (1-4) and reliability (5-9) of websites.

Question	
Number	Question framing
1	Is the site design clear and transparent?
2	Is the site design consistent from one page to the other?
3	Can users find what they need on the site?
4	Is the format of information clear and appropriate for the audience?
5	Is it clear who has developed the website and what their objectives are?
6	Does the site report a robust quality control procedure?
7	Is the page content checked by an expert?
8	Is the page updated regularly?
9	Does the page cite relevant sources where appropriate?

**Table 2.** Details of the sites analyzed.

Number	Website	Name	Profession	Type of information
1	jdnheidi.com	M	Maxillofacial surgeon	Orthognathic Surgery
2	drjui.com	M	Maxillofacial surgeon	Orthognathic Surgery
3	aoms.co.nz	M	Maxillofacial surgeon	Orthognathic Surgery
4	orthocj.com	M	Orthodontist	Orthognathic Surgery
5	fairfaxoralsurgery.com	M	Maxillofacial surgeon	Orthognathic Surgery
6	aaoms.org	N/M	N/M	Orthognathic Surgery
7	ehow.com	M	Professional media	General
8	omsaofwm.com	M	Maxillofacial surgeon	Orthognathic Surgery
9	surgicalarts.net	M	Maxillofacial surgeon	Orthognathic Surgery
10	soms.com	M	Maxillofacial surgeon	Orthognathic Surgery
11	smilesolutions.com	M	Maxillofacial surgeon	Orthognathic Surgery
12	drwmcdonald.com	M	Maxillofacial surgeon	Orthognathic Surgery
13	uihealthcare.com	M	Maxillofacial surgeon	Orthognathic Surgery
14	surgery-guide.com	N/M	N/M	Orthognathic Surgery
15	surgery.med.umich.edu	N/M	N/M	Orthognathic Surgery
16	cirugiafacial.com	M	Cosmetic Surgeon	General
17	orthognathic-surgery.org	N/M	N/M	Orthognathic Surgery
18	ckjohnson.com	M	Maxillofacial surgeon	Orthognathic Surgery
19	omswinnebago.com	M	Maxillofacial surgeon	Orthognathic Surgery
20	omfsurgery.com	M	Maxillofacial surgeon	Orthognathic Surgery
21	nguyenorthodontics.net	M	Orthodontist	Orthognathic Surgery
22	orthognathicsurgerycost.org	M	Orthodontist	Orthognathic Surgery
23	cosmeticdentistryguide.co.uk	M	Maxillofacial surgeon	Orthognathic Surgery
24	cosmeticvacations.com	N/M	N/M	Orthognathic Surgery
25	la-coms.com	M	Maxillofacial surgeon	Orthognathic Surgery
26	oregonoralsurgery.com	M	Maxillofacial surgeon	Orthognathic Surgery
27	snorenet.com	M	Maxillofacial surgeon	Orthognathic Surgery
28	atl-ofs.com	M	Maxillofacial surgeon	Orthognathic Surgery
29	cmsllc.com	M	Maxillofacial surgeon	Orthognathic Surgery

M = mentions the author; N/M = does not mention the author.

## Precision

With regard to precision, and taking the criteria of duration, etiology and pain management into account, the following percentages were achieved: 24.14% of websites did not address the subject of pain; 34.49% of them discussed about pain based on one criterion, only; 17.25% discussed about pain based on two criteria; in 10.35% all three criteria were discussed with important omissions; in 13.80% all three criteria were discussed with minor omissions; and none of the researched websites conducted a complete discussion. The mean for all websites was 30.34. Standard deviation (SD) was 27.05.

## Readability

Readability assessment revealed that FRES ranged between 27.6 and 71.5. The mean FRES value for all websites was 53.96. Therefore, they were all considered very difficult to read, and accounted for university graduate or undergraduate reading level.

## Accessibility

Accessibility of all websites was considered medium (82.93 ± 10.86). Ten web pages were found to have high accessibility results (> 90%). The lowest result was found in two websites (63%). Six web pages had the highest accessibility results (94 %).

## Usability

Mean usability of all websites was 53.31 ± 14.54. Two websites had the highest results (75%), in both of them information was provided by an oral and maxillofacial surgeon. The lowest result was found in one website of unknown authorship (17%).

## Reliability

Reliability of all websites examined in this study was considered low (37.31 ± 13.62). The highest result was found in two websites both edited by oral and maxillofacial surgeons (53%). In one of them, reliability was 0 and the author was not identified.

## LIDA results

The overall mean validation for the 29 websites was 65.10 ± 9.43, thereby indicating them to be of medium quality (Table 4). LIDA percentage results ranged from 37 to 74. The highest score was attributed to a website that discussed orthognathic surgery and whose author was an oral and maxillofacial surgeon. The lowest score was attributed to a website discussing on orthogenetic surgery, but which did not touch on the subject of pain and did not present the author's name and profession.

**Table 3.** Evaluation of the websites in terms of precision, Flesch Easy Reading Scale (FRES), accessibility, usability, reliability and LIDA (* = the maximal score possible; between brackets = the mean corresponding percentages).

Number	Website	Precision 5*	FRES 100*	Accessibility 54*	Usability 12*	Reliability 30*	LIDA 96*
1	jdnheidi.com	2 (40)	44	38 (70)	6 (50)	10 (33)	54 (56)
2	drjui.com	1 (20)	71.5	51 (94)	8 (67)	14 (47)	73 (76)
3	aoms.co.nz	4 (80)	43.2	45 (83)	9 (75)	12 (40)	66 (69)
4	orthocj.com	0 (0)	44.9	44 (81)	8 (67)	16 (53)	68 (71)
5	fairfaxoralsurgery.com	2 (40)	69	42 (78)	8 (67)	14 (47)	64 (67)
6	aaoms.org	2 (40)	39	47 (87)	6 (50)	14 (47)	67 (70)
7	ehow.com	1 (20)	70.4	46 (85)	8 (67)	8 (27)	62 (65)
8	omsaofwm.com	4 (80)	58.2	45 (83)	6 (50)	12 (40)	63 (66)
9	surgicalarts.net	1 (20)	62.4	50 (93)	8 (67)	12 (40)	70 (73)
10	soms.com	1 (20)	30.5	49 (91)	4 (33)	12 (40)	65 (68)
11	smilesolutions.com	1 (20)	52.1	45 (83)	8 (67)	14 (47)	67 (70)
12	drwmcdonald.com	4 (80)	62.9	35 (65)	8 (67)	16 (53)	59 (61)
13	uihealthcare.com	0 (0)	55.1	32 (59)	6 (50)	14 (47)	52 (54)
14	surgery-guide.com	0 (0)	35.3	34 (63)	3 (25)	0 (0)	37 (39)
15	surgery.med.umich.edu	2 (20)	69.2	34 (63)	2 (17)	4 (13)	40 (42)
16	cirugiafacial.com	1 (20)	27.6	39 (72)	6 (50)	12 (40)	57 (59)
17	orthognathic-surgery.org	0 (0)	43.5	45 (83)	5 (42)	2 (7)	52 (54)
18	ckjohnson.com	1 (20)	42.8	44 (81)	6 (50)	12 (40)	62 (65)
19	omswinnebago.com	2 (40)	61.9	51 (94)	8 (67)	12 (40)	71 (74)
20	omfsurgery.com	1 (20)	70.1	51 (94)	7 (58)	12 (40)	70 (73)
21	nguyenorthodontics.net	0 (0)	32.3	48 (89)	4 (33)	12 (40)	64 (67)
22	orthognathicsurgerycost.org	4 (80)	51.6	48(89)	6 (50)	12 (40)	66 (69)
23	cosmeticdentistryguide.co.uk	0 (0)	48.5	49 (91)	5 (42)	12 (40)	66 (69)
24	cosmeticvacations.com	1 (20)	46.7	47 (87)	6 (52)	2 (7)	55 (57)
25	la-coms.com	3 (60)	52.2	51 (94)	7 (58)	14 (47)	72 (75)
26	oregonoralsurgery.com	3 (60)	67.3	51 (94)	7 (58)	12 (40)	70 (73)
27	snorenet.com	3 (60)	70.8	51 (94)	9 (75)	14 (47)	74 (77)
28	atl-ofs.com	1 (20)	56.5	39 (72)	6 (50)	12 (40)	57 (59)
29	cmsllc.com	0 (0)	46.5	50 (93)	5 (42)	12 (40)	67 (70)

**Table 4.** Mean and standard deviation (SD) values for the categories assessed - Percentage of score (n = 29).

	Precision	FRES	Accessibility	Usability	Reliability	LIDA
Mean	30.34	53.96	82.93	53.31	37.31	65.10
SD	27.05	12.26	10.86	14.54	13.62	9.43

## DISCUSSION

The search for information on the internet enables an interchange of experiences among patients with similar health problems.^16^ The worldwide dissemination of this practice offers the possibility of globally exchanging knowledge.^17^ However, the quality of health information available on the internet is doubtful, since a great deal of information is incomplete or inadequate.^7^^,^ 18 ^-^ 21 Following this line of reasoning, it is reasonable to think: What is the status of information available to patients who will be submitted to orthognathic surgery?

It is known that many doubts and uncertainties surround patients who will be submitted to orthognathic surgery. Undoubtedly, post-operative pain is the most prevalent doubt, and is also the main reason for patients giving up this procedure. Nevertheless, how are these patients being informed about post-operative pain? In an endeavor to elucidate this question, the present study assessed the quality of information available on the internet about post-operative pain after orthognathic surgery.

Investigating the quality of this type of information available on the internet is of paramount importance because any person, group of interests, company or institution may publish any kind of information without being subject to peer reviews and without taking into account that many medical questions are difficult to understand and not all levels of education are able to understand information that is available.^22^

A major concern about assessment criteria arose while the present study was being conducted. These criteria included precision, integrity, ease of reading, disclosure and references.^23^^,^ 24 ^,^ 25 The five main browsers providing access to popular search engines in the world were used,: Google, Bing, Yahoo!, Ask and AOL, which according to up-to-date statistical data, respectively represent 65.9%, 15.1%, 14.5%, 2.9% and 1.6% of most researches conducted in the United States.^6^^,^ 26

Studies assessing the nature of pain after orthognathic surgery are not commonly found, except for one study that generally analyzed the subject.^27^ There was considerable divergence in the information found in comparison to scientific evidence, textbooks, primary literature and what is experienced in clinical practice, thereby compromising precision of information. As regards to precision of the websites, 75.88 % discussed two or fewer topics about pain, diverging from the study about pain during orthodontic treatment conducted by Livas et al^6^ in which the percentage was 80%. The mean criteria verification was 1.55, thus indicating that the information found was incomplete. 

In writing the texts, the authors did not take into consideration that they should be understandable by all socioeconomic and educational levels, thereby compromising understanding by certain groups.^22^^,^ 28 The mean FRE for all websites was 53.96, while in the study by Patel and Cobourne^22^ the average reading ease scale was 58.3. In the present study, the texts available on the websites were evaluated as being very difficult to read.

One of the assessment tools used was the LIDA instrument which consists of a widely used set of criteria used to assess the value of health material available on the internet. The mean global validation for the 29 websites provided by LIDA instrument was 65.10, thereby indicating medium quality. Accessibility of all websites was considered average. The mean usability of all websites was 53.31, varying among those more and less frequently accessed. Reliability of all websites examined in this study was considered low, given the incomplete sets of information provided.

The continuous development of information on pain after orthognathic surgery available on electronic resources and communities would contribute towards reducing anxiety and providing greater confidence for potential candidates of orthosurgical treatment. What should be done is to create criteria to analyze health information available on the internet, so that after the material has been analyzed, it would have permission to be published for use.

While this does not occur, internet users must be aware of the limitations in seeking counseling on dental procedures online. Additionally, they must allow themselves to be guided by specialists in valid web databases.

## CONCLUSION

Based on the results of this study it is reasonable to conclude that:

" The quality of information found in the evaluated websites was considered poor.

" Articles on pain after orthognathic surgery found in websites on the internet are poorly written and not reliable.

" The websites were also considered as divulging incomplete data that demand reading skills of university graduates or undergraduates.
